# Transapical extirpation of a left ventricular thrombus in takotsubo cardiomyopathy

**DOI:** 10.1186/1749-8090-8-135

**Published:** 2013-05-26

**Authors:** Ryo Suzuki, Tomoaki Kudo, Hiroshi Kurazumi, Masaya Takahashi, Bungo Shirasawa, Akihito Mikamo, Kimikazu Hamano

**Affiliations:** 1 Department of Surgery and Clinical Science, Division of Cardiac Surgery, Yamaguchi University Graduate School of Medicine, 1-1-1 Minami-Kogushi, Ube, Yamaguchi 755-8505, Japan

**Keywords:** Thrombosis, Takotsubo cardiomyopathy, Transapical extirpation

## Abstract

A 58-year-old Japanese female was referred to our hospital. Although the electrocardiogram showed ST elevation, coronary angiography showed intact coronary artery. We diagnosed Takotsubo cardiomyopathy and a left ventricular thrombus. Anticoagulation was administered; however, the left ventricular thrombus had become mobile and protrusive. We extirpated the left ventricular thrombus via trans-apical approach. Left ventricular thrombus is rare in Takotsubo cardiomyopathy, but these patients are at a higher risk of thromboembolism, especially if the thrombi are mobile and protruding.

## Background

Takotsubo cardiomyopathy is a disease characterized by a reversible left ventricular wall motion abnormality. The prognosis is favorable if there is normalization of the wall motion abnormalities within a few weeks. Although some investigators have reported cases of thromboembolic complications, such as cerebral infarction with a left ventricular thrombus, no published guidelines exist for the management of Takotsubo cardiomyopathy with a left ventricular thrombus. We herein report a case of Takotsubo cardiomyopathy with a left ventricular thrombus that was successfully removed during surgery.

## Case presentation

**Figure 1:**
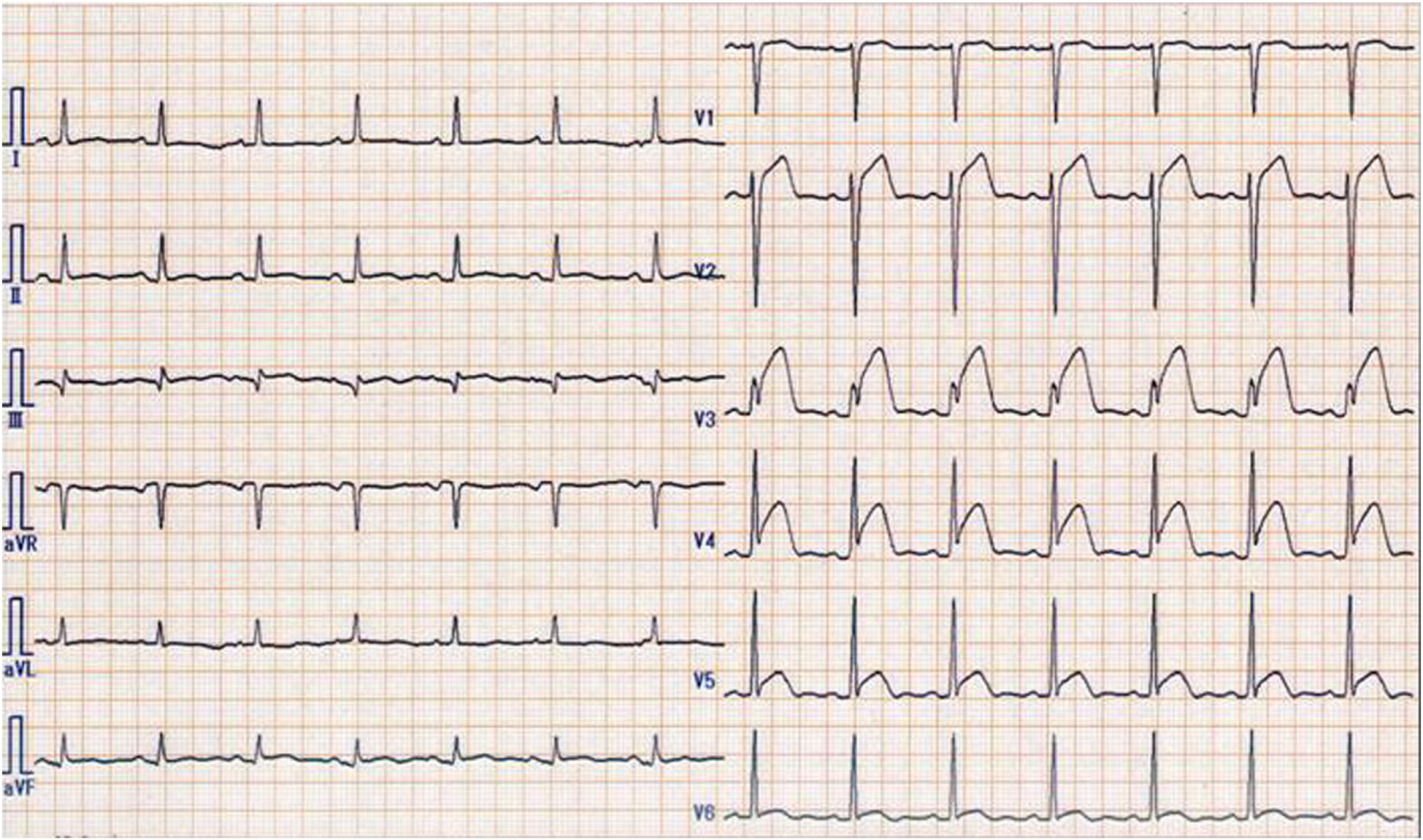
The electrocardiogram showed ST elevation in leads V2–V6.

A 58-year-old Japanese female with no previous cardiac history any other medical history experienced chest discomfort. She was seen at a nearby clinic the next day, and was referred to our hospital for probable acute coronary syndrome. The electrocardiogram showed ST elevation in leads V2–V6 (Figure 1). The echocardiographic examination showed a dilated left ventricle with a severely reduced ejection fraction, but preserved basal contractile function. Coronary angiography showed no coronary artery disease. Based on these findings, we diagnosed the patient with Takotsubo cardiomyopathy. The echocardiographic examination performed on the same day showed a thrombus in the apex of the left ventricle (Additional file 1). Therefore, heparin was immediately administered intravenously and warfarin therapy was started. On hospital day 7, echocardiography showed that the left ventricular apical asynergy had almost disappeared, but the size of the left ventricular thrombus had not changed. Furthermore, the thrombus had become mobile and protrusive (Additional file 2). We planned extirpation of the left ventricular thrombus, because we considered the thromboembolic risk to be high.

**Figure 2:**
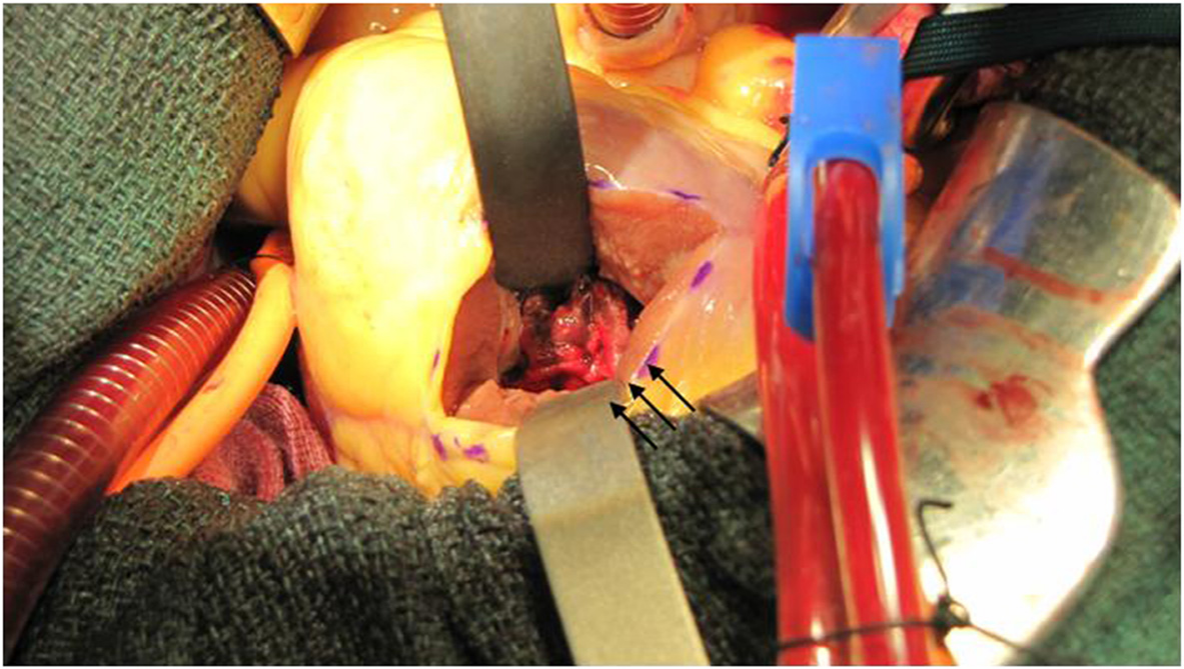
There were mural thrombus (black arrow).

The patient was transferred to the operating room. Under cardiopulmonary bypass and cardiac arrest using tepid blood cardioplegia, the apex of the left ventricle was incised approximately 2 cm away from and parallel to the left anterior descending artery. The incision was extended anterior and posterior of the apex by 2 cm to expose the fragile thrombus (Figure 2). The thrombus was extirpated, and the left ventricular cavity was irrigated. The ventriculotomy was repaired with double felt strips. The postoperative course was uneventful.

## Discussion

Takotsubo cardiomyopathy is described as a reversible left ventricular apical wall motion abnormality with chest symptoms. Although ST segment elevation is present in the electrocardiogram and there is an abnormal pattern of ventricular contraction, there is a lack of significant coronary artery stenosis [1]. The prognosis of Takotsubo cardiomyopathy is usually good, but serious complications, such as thromboembolism, can occur [2]. It is well known that left ventricular thrombi often occur after a myocardial infarction. Patients with anterior myocardial infarction are at an especially high risk of left ventricular thrombus development. The formation of a thrombus can be consequence of increased infarct size, pump failure, severe apical asynergy and decreased global left ventricular function [3]. In cases of Takotsubo cardiomyopathy, the ventricular dysfunction extends beyond a single coronary artery region. A left ventricular apical dysfunction resembling that of an aneurysm can occur. This is thought to be a possible cause of a left ventricular thrombus. Therefore, anticoagulation therapy is recommended for patients with Takotsubo cardiomyopathy. However, even under anticoagulation therapy, some investigators reported the incidence of left ventricular thrombus in the setting of Takotsubo cardiomyopathy to be 5.3–8% [4].

It is unknown whether thromboembolism is common in cases of Takotsubo cardiomyopathy. In cases of myocardial infarction, some authors have suggested that the thromboembolic risk was significantly higher in cases with thrombus protrusion and mobility [5]. Thus, surgical extirpation is recommended to prevent thromboembolism. However, the area of myocardial infarction does not change the location where the thrombi generally occur. However, the wall abnormalities associated with Takotsubo cardiomyopathy are usually resolved within a few weeks. Upon normalization of the wall motion, if the left ventricular thrombus remains, the thromboembolic risk rate is high. In fact, some investigators have reported that thromboembolic complications occurred in 20–33.3% of the cases of Takotsubo cardiomyopathy [2-4]. Therefore, we performed surgical extirpation of the left ventricular thrombus, because it had morphologic features suggesting a high risk of thromboembolism. Seitz MJ and colleagues reported the case of Takotsubo cardiomyopathy related left ventricular apical thrombus requiring surgery [6]. Also, they concluded surgical management of mobile and pedunculated left ventricular thrombus related to Takotsubo cardiomyopathy is warranted.

## Conclusion

Early surgical extirpation is strongly recommended when a left ventricular thrombus in patients with Takotsubo cardiomyopathy becomes mobile and protrusive because of the high risk of thromboembolism.

## Consent

Written informed consent was obtained from the patient for publication of this Case report and any accompanying images. A copy of the written consent is available for review by the Editor-in-Chief of this journal.

### Ethical approval

All participants of this study were provided appropriate informed consent, and the ethics review boards of Yamaguchi University Hospital approved the study design.

## Competing interests

The authors declare that they have no competing interests.

## Authors’ contributions

RS: main author wrote the paper. AM: performed the surgery and helped to draft manuscript. TK and HK and MT and BS: helped to draft manuscript. KH: reviewed the manuscript, and is the corresponding author. All authors read and approved the final manuscript.

## Supplementary Material

Additional file 1 **The apical long-axis view demonstrated a thrombus.** There was a mural thrombus. Click here for file

Additional file 2 Echocardiography seven days later showed a mobile and protrusive thrombus. Click here for file
